# Book review of *Health and Welfare of Captive Reptiles*, Second Edition – Editors’ Note

**DOI:** 10.1017/awf.2025.10033

**Published:** 2025-08-13

**Authors:** Birte L. Nielsen, T. Bas Rodenburg

**Affiliations:** 1 Universities Federation for Animal Welfare, Hertfordshire, UK; 2 Department Population Health Sciences, Utrecht University, The Netherlands

It has been suggested to the Editors that the following sentences in this book review may be open to misinterpretation:

*“I approached this review with a degree of apprehension, knowing the connection of some authors with the Animal Rights movement, who openly admit to an agenda of eliminating all reptile-keeping. Such negative views are not helpful in encouraging improvements in animal welfare and their absence from almost all chapters is a relief”.*

The author of the review, Dr Frances Baines, wishes to confirm, as is apparent when read in the wider context of the review, that these sentences refer to the generality of authors, not to those specifically associated with the title or the particular chapter she was reviewing.

The authors of Chapter 16 (Dr Mike Jessop, Dr Anthony Pilny, Dr Clifford Warwick, and Dr Martin Whitehead) wish to assert that they are not, and have not been, affiliated with the Animal Rights movement.
